# Phylogenetic position and taxonomy of *Kusaghiporia usambarensis* gen. et sp. nov. (Polyporales)

**DOI:** 10.1080/21501203.2018.1461142

**Published:** 2018-04-15

**Authors:** Juma Mahmud Hussein, Donatha Damian Tibuhwa, Sanja Tibell

**Affiliations:** a Institute of Organismal Biology, Evolutionary Biology Centre, Uppsala University, Uppsala, Sweden; b Department of Molecular Biology and Biotechnology, College of Natural & Applied Sciences, University of Dar es Salaam, Dar es Salaam, Tanzania

**Keywords:** *Kusaghiporia*, molecular phylogeny, polyporales, Tanzania, taxonomy, Usambara

## Abstract

A large polyporoid mushroom from the West Usambara Mountains in North-eastern Tanzania produces dark brown, up to 60-cm large fruiting bodies that at maturity may weigh more than 10 kg. It has a high rate of mycelial growth and regeneration and was found growing on both dry and green leaves of shrubs; attached to the base of living trees, and it was also observed to degrade dead snakes and insects accidentally coming into contact with it. Phylogenetic analyses based on individual and concatenated data sets of nrLSU, nrSSU and the RPB2 and TEF1 genes showed it, together with *Laetiporus, Phaeolus, Pycnoporellus* and *Wolfiporia*, to form a monophyletic group in *Polyporales*. Based on morphological features and molecular data, it is described as *Kusaghiporia usambarensis*.

## Introduction

*Polyporales* is an order of fungi in Basidiomycota containing more than 1800 species in 216 genera and 13 families (Kirk et al. 2008). However, Justo et al. (2017) recognised 37 families in *Polyporales*. Seven clades have been recognised in *Polyporales*: the “antrodia”; “core polyporoid”; “residual polyporoid”; “phlebioid”; “tyromyces”; “gelatoporia” and “fragiliporia” clades (Binder et al. 2013; Zhao et al. 2015).

The “antrodia clade” was first identified by Hibbett and Donoghue (2001) and currently more than 26 genera are recognised in this clade (Ortiz-Santana et al. 2013). Members in the “antrodia clade” are of economic importance as a source of food, and also of pharmaceutical and biotechnological products. However, it also contains species that are plant pathogens detrimental to forests and forest plantations (Dai et al. 2007; Banik et al. 2010). The “antodia clade” is morphologically diverse and includes species that have resupinate, stipitate or pileatebasidiomata that are either annual or perennial; the hyphal system is monomitic, dimitic or trimitic; the basidiospores are hyaline thin- to thick-walled, subglobose to cylindricaland they cause brown rots (Ryvarden and Melo 2014).

The “antrodia clade” has been widely studied and additional genera have been suggested to belong there. Recent taxonomic and phylogenetic studies, including that of Lindner and Banik (2008), have presented molecular phylogenies of the clade. In a study of *Laetiporus* and other polypores, Banik et al. (2010) inferred relationships among North American and Japanese *Laetiporus* isolates; Ortiz-Santana et al. (2013) presented a phylogenetic overview of the “antrodia clade” and Binder et al. (2013) used genomic data and a six-gene data set for evaluating phylogenetic relationships in *Polyporales*. Further studies include those of Han et al. (2014) in which two new species of *Fomitopsis* from China were described, and Zhao et al. (2015) used a multi-gene dataset to support the recognition of *Fragiliporiaceae*, a new family of *Polyporales*. Han et al. (2016) offered a study of the phylogeny of the brown-rot fungi, including *Fomitopsis* and related genera, while Justo et al. (2017) revised the phylogeny of *Polyporales* at family-level.

A mushroom locally known as “Kusaghizi” has a long tradition of being used as food by local communities in the Usambara mountains as first reported by Powell et al. (2013). In a study by Juma et al. (2016), which assessed antioxidant activities of saprobic mushrooms from Tanzania, “Kusaghizi” was included, but neither the study by Powell et al. nor that of Juma et al. reported a scientific name for “Kusaghizi”. Here we aim to describe this species and infer its phylogenetic position.

## Material and methods

### Material

Material of the fungus locally named “Kusaghizi” was collected during the rainy seasons in February 2016 and March 2017 close to the villages Bungu, Buti and Makuri in the Usambara Mountains. These villages are located in the Korogwe District of the Tanga region, Tanzania. The west Usambara Mountains are part of the “Eastern Arc” of ranges in eastern Tanzania, from the Taita Hills in Kenya to the Udzungwa Mountains in southern Tanzania. The samples were examined in a fresh condition for macro-morphological features including colour changes upon cut, bruising and exposure to air. A fruit body was divided into two parts; one was sun dried for 5 days while the remaining part was stored in a freezer at −20°C for further investigations.

### Morphological characterisation

Basidioma colours of the holotype were indicated according to Kornerup and Wanschern (1967). Photographs of the fruit body were taken before and after removing it from its substrate. Microscopic characterisation was done from preparations of a rehydrated specimen sectioned with a freezing microtome and stained with Lactic Blue, or treated with 10% KOH and Melzer’s reagent.

A total of 40 mature basidiospores were randomly selected and measured. Statistical averages were used to estimate the observed features as follows: *A*_L_ = mean spore length (arithmetic mean of the length of spores); *A*_W_ = mean spore width (arithmetic mean of the width of spores); *Q* = *A*_L_/*A*_W_ ratio; *n* (*a*/*b*) = number of spores (*a*) measured from given number (*b*) of specimen. Melzer’s reagent was used where IKI+ = Melzer’s reagent positive; IKI− = both inamyloid and indextrinoid.

### DNA extraction, amplification and sequencing

Total genomic DNA was extracted from both fresh and dried material and kept at −20°C following the protocol of the plant Genomic DNA extraction kit (E.Z.N.A. Fungal DNA Mini Kit Protocols). Diluted samples (10^–1^) of DNA were used for PCR amplification of the nrLSU, nrSSU, RPB2 and TEF1. Primers LR0R, LR7, LR5 were used for nrLSU (Vilgalys and Hester 1990), and PNS1 and NS41 for nrSSU (Hibbett 1996). PCR conditions for nrLSU and nrSSU were: initial denaturation for 4 min at 95°C, followed by 35 cycles of 1 min at 94°C, 1 min at 54°C, 45 s at 72°C, and a final elongation for 5 min at 72°C. The RPB2 region was amplified using degenerated primers fRPB2-5f and RPB2-7.1R (Matheny 2005). For amplification of TEF1the EF1-983Fand EF1-1567R primers were used (Rehner and Buckley 2005). Touchdown PCR was used with an initial annealing temperature of 66°C following the protocol of Rehner and Buckley (2005). The PCR products were visualised by electrophoresis on 1.5% agarose gels. Products were purified using Illustra™ ExoStar buffer diluted 10×, following the manufacturer’s protocol. Sequencing was carried out by Macrogen.

### Data analyses

Sequences from GenBank were selected based on their quality and with an intention of wide coverage of Polyporales and the “antrodia clade” as in Zhao et al. (2015) and Han et al. (2016) respectively. The sequences produced in this study were aligned along with those downloaded from GenBank (Table 1) using MAFFT v. 7 (http://mafft.cbrc.jp/alignment/server/) and manually adjusted using AliView (Larsson 2014). Ambiguously aligned regions were excluded from the analyses. For RPB2 and TEF1 only coding parts of the sequences were used for the analyses. The concatenated data matrix of *Polyporales*and the “antrodia clade” contained 4940 and 3760 unambiguously aligned sites respectively. All alignments were based on the nucleotide sequences with each gene analysed separately.

**Table 1.** Species, collection and GenBank accession number of sequences used in this study. New sequences in bold.

GenBank accession						
Species name	Collection number	nrLSU	nrSSU	tef1	rpb2	References
*Albatrellus higanensis*	AFTOL-ID 774	AY684166	AY707091	DQ059049	AY780935	Matheny et al. (2007)
*Amyloporia carbonica*	Cui 12212	KR605755	KR605917	KR610745	—	Han et al. (2016)
*A. xantha*	Cui 11677	KR605757	KR605919	KR610747	KR610837	Han et al. (2016)
*Antrodia albida*	FP 105979	EU232272	AY336777	—	DQ491387	Kim et al. (2007)
*A. heteromorpha*	Dai 12755	KP715322	KR605908	KP715336	KR610828	Chen and Cui (2016)
*A. macra*	Eriksson 1967	R605749	KR605909	KR610739	—	Han et al. (2016)
*A. serialis*	Cui 10519	KP715323	KR605911	KP715337	KR610830	Han et al. (2016)
*A. serpens*	Dai 7465	KR605752	KR605913	KR610742	KR610832	Han et al. (2016)
*A. tanakae*	Cui 9743	KR605753	KR605914	KR610743	KR610833	Han et al. (2016)
*A. variiformis*	CBS 309.82	AY515344	JF972578	—	DQ491391	Kim et al. (2007)
*Bjerkandera adusta*	HHB-12826-Sp	MF115840	DQ060085	—	KP134913	Floudas and Hibbett (2015)
*Buglossoporus eucalipticola*	Dai 13660	KR605747	KR605906	KR610736	KR610825	Han et al. (2016)
*B. quercinus*	JV 1406/1	KR605740	KR605899	KR610730	KR610820	Han et al. (2016)
*Climacodon septentrionalis*	AFTOL-ID 767	AY684165	AY705964	AY885151	AY780941	
*Coriolopsis polyzona*	Cui 11040	KR605767	KR605932	KR610760	KR610849	Han et al. (2016)
*Crustoderma flavescens*	HHB-9359-Sp	KC585150	—	—	—	Ortiz-Santana et al. (2013)
*C. longicystidia*		AY219388	—	—	—	
*C. resinosum*	L-10631-Sp	KC585155	—	—	—	Ortiz-Santana et al. (2013)
*Daedalea africana*	O 15372	KP171216	KR605871	KR610704	KR610795	Han et al. (2015)
*D. allantoidea*	Dai 13612A	KR605734	KR605892	KR610723	KR610813	Han et al. (2016)
*D. radiata*	Cui 8575	KP171233	KR605888	KR610720	KR610811	Han et al. (2015)
*D. quercina*	Dai 12152	KP171229	KR605886	KR610717	KR610809	Han et al. (2015)
*Fibroporia albicans*	Dai 10595	KR605759	—	—	—	Chen et al. (2016)
*F. radiculosa*	Cui 11404	KR605760	KR605922	KR610750	KR610840	Chen et al. (2016)
*Fomitopsis durescens*	O 10796	KF937294	KR605834	KR610669	KR610766	Han et al. (2014)
*F. ibericus*	O 10810	KR605710	KR605842	KR610676	KR610771	Han et al. (2016)
*F. palustris*	Cui 7597	KP171236	KR605854	KR610687	KR610778	Han et al. (2015)
*F. pinicola*	Cui 10312	KR605720	KR605856	KR610689	KR610780	Han et al. (2016)
*Fragifomes niveomarginata*	Cui 10108	KR605717	KR605851	KR610684	KR610776	Han et al. (2016)
*Fragiliporia fragilis*	Dai 13559	KJ734265	—	KJ790246	KJ790249	Zhao et al. (2015)
*F. fragilisi*	Dai 13080	KJ734264	—	KJ790245	KJ790248	Zhao et al. (2015)
*Ganoderma lucidum*	BEOFB434	X78776	KY464926	KX371599	KX371601	
*G. tsugae*	AFTOL-ID 771	AY684163	AY705969	DQ059048	DQ408116	Matheny et al. (2007)
*Heterobasidion annosum*	06129/6	KJ583225	U59072	AB472644	KJ651728	Chen et al. (2016)
*Junghuhnia nitida*	KHL 11903	EU118638	AF082685	JN710721	KP134964	Larsson (2007)
*Kusaghiporia usambarensis*JMH 01	**J. Hussein 01/16**	**MH010044**	**MH010046**	**MH048871**	**MH048870**	**This study**
*K. usambarensis*JMH 02	**J. Hussein 01/17**	**MH010045**	—	**MH048869**	—	**This study**
*Laetiporus cincinnatus*	JV 0709/168J	KF951305	KX354517	KY886787	KY886801	Song et al. (2014)
*L. persicinus*	HHB9564	EU402513	—	—	—	Lindner and Banik (2008)
*L. persicinus*	RLG14725	EU402512	—	—	—	Lindner and Banik (2008)
*L. sulphureus*	Dai 12154	KF951302	KR605924	KR610752	KR610841	Song et al. (2014)
*Laricifomes officinalis*	JV 0309/49-J	KR605764	KR605929	KR610757	KR610846	Han et al. (2016)
*Phaeolus schweinitzii* AFTOL 702	AFTOL-ID 702	AY629319	AY705961	DQ028602	DQ408119	Matheny et al. (2007)
*P. schweinitzii* Dai 8025	Dai 8025	KC585197	KX354553	KX354686	LN714690	Song and Cui (2017)
*Phanerochaete chrysosporium*	BKM-F-1767	GQ470643	KJ606692	HQ188380	KP134954	Wu et al. (2010)
*Phlebia radiata*	AFTOL-ID 484	AF287885	AY946267	AY885156	AY218502	Hibbett et al. (2000)
*Piptoporellus hainanensis*	Dai 13714	KR605745	KR605904	KR610735	KR610824	Han et al. (2016)
*P. soloniensis*	LY BR 5463	KR605744	KR605903	KR610734	KR610823	Han et al. (2016)
*Polyporus arcularius*	DSH92132	KP283522	KX549013	—	AB368139	Seelan et al. (2015)
*P. squamosus*	AFTOL-ID 704	AF135181	AY705963	DQ028601	DQ408120	Matheny et al. (2007)
*Polyporales* sp. Kusaghizi IJV 01	IJV40-1	KM593894	—	—	—	
*Polyporales* sp. Kusaghizi IJV 02	IJV40-2	KM593895	—	—	—	
*Postia duplicata*	Dai 13411	KJ684976	KR605928	—	KR610844	Ll and Bk (2014)
*Pycnoporellus alboluteus*	HHB-17598-Sp	KC585216	—	—	—	Ortiz-Santana et al. (2013)
*P. fulgens*	CA-20	KC585218	—		—	Ortiz-Santana et al. (2013)
*Pycnoporus* sp.	ZW02.30	AY684160	GU182936	DQ028600	DQ408121	Matheny et al. (2007)
*Steccherinum ochraceum*		EU118670	—	JX109893	JN710738	Larsson (2007)
*Stereum hirsutum*	FP-91666	AY039330	AF026588	XM007298185	AY218520	Wu et al. (2001)
*Trametes suaveolens*	Cui 11586	KR605766	KR605931	KR610759	KR610848	Han et al. (2016)
*Trametes versicolor*	Dai10998	KC848354	KR261697	JN164878	DQ408125	Justo and Hibbett (2011)
*Ungulidaedalea fragilis*	Cui 10919	KF937290	KR605840	KR610674	KR610770	Han et al. (2014)
*Wolfiporia cocos* EF 397599	18176	KC585233	—	—	—	Ortiz-Santana et al. (2013)
*W. cocos voucher* CBK 1	CBK-1	KX354689	KX354690	KX354688	KX354685	Song and Cui (2017)
*W. cartilaginea*	13121	KC585405	—	—	—	Ortiz-Santana et al. (2013)
*W. dilatohypha*	FP-72162-R	KC585235	—	—	—	Ortiz-Santana et al. (2013)

Single-gene analyses were performed to detect significant conflicts among datasets. A conflict among single-locus datasets (nrLSU, nrSSU, RPB1, TEF1) was considered significant if a well-supported monophyletic group, for example posterior probability (PP) ≥ 0.95, was found to be well supported as non-monophyletic when different loci were used. No significant incongruence among the single-gene trees was detected (Supplementary Figures S1A, S1B S1C and S1D), hence the four matrices were concatenated.

Further analyses were carried out after concatenation using Sequence Matrix (Vaidya et al. 2011).

The best-fit model of DNA evolution for the analyses, for both individual codon positions and genes, was obtained using the Akaike Information Criterion as implemented in MrModeltest 2.3 (Nylander 2004). For the *Polyporales* dataset the GTR+I + G model was employed across sites for nrLSU, nrSSU, and for the 1st and 2nd codon for RPB2. For the 1st and the 2nd codon for TEF1 the model F81 + I + G was applied, while GTR + G was implemented for both the 3rd codon of RPB2 and TEF1. For the “antrodia clade” dataset the GTR+I + G model was employed across sites for nrLSU, nrSSU, for all three codons for RPB2, and the 2nd codon for TEF1. For the 1st codon for TEF1 the F81 + I model was applied while the HKY + I + G model was implemented for the 3rd codon for TEF1. Bayesian Inference was conducted with MrBayes 3.2.6, and branch support was estimated by PP (Ronquist and Huelsenbeck 2003). Four Markov chains were run for 2 runs from random starting trees for 10 million generations, trees were sampled every 100 generations and 25% were discarded as burn-in.

Maximum likelihood estimates were carried out by RAxML v.8.2.10 using the GTR +G + I model of site substitution (Stamatakis 2014). The branch support was obtained by maximum likelihood bootstrapping (MLbs) of 1000 replicates (Hillis and Bull 1993).

Bayesian PPs ≥ 0.95 (Alfaro et al. 2003), and MLb ≥ 70% were considered to be significant. Sequence alignments and phylogenetic trees were deposited in TreeBase, submission ID: (http://purl.org/phylo/treebase/phylows/study/TB2:S223838).

## Results

### Phylogenetic analyses

**Figure 1.**
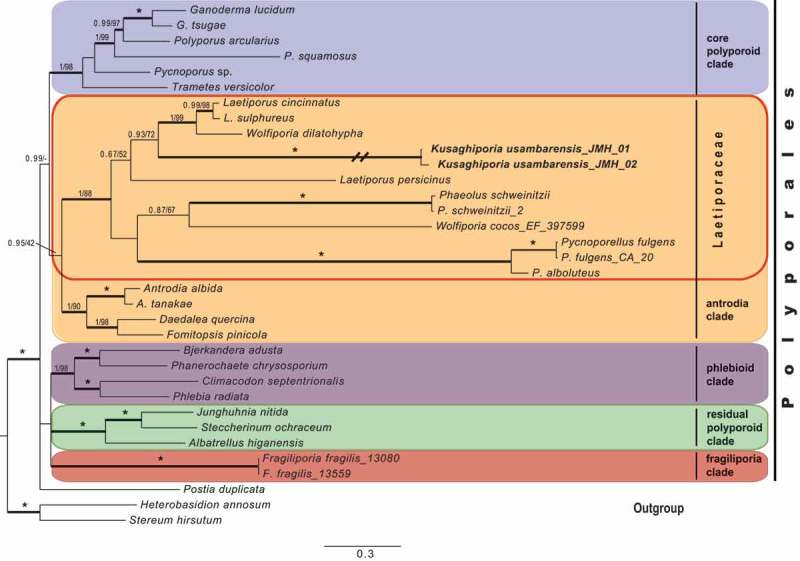
Phylogenetic relationships among *Kusaghiporia usambarensis* and allied taxa in *Polyporales*, based on Bayesian and ML analyses of concatenated nrLSU, nrSSU, RPB1 and TEF1 datasets. The tree was rooted using two species from Russulales (*Heterobasidion annosum* and *Stereum hirsutum*). The two support values associated with each internal branch correspond to PPs and MLbs proportions, respectively. Branches in bold indicate a support of PP ≥ 0.95 and MLbs ≥ 70%. An asterisk on a bold branch indicates that this node has a support of PP = 1.0 and MLbs = 100. The branch with double-slash is shortened. Clade names follow Zhao et al. (2015).

**Figure 2.**
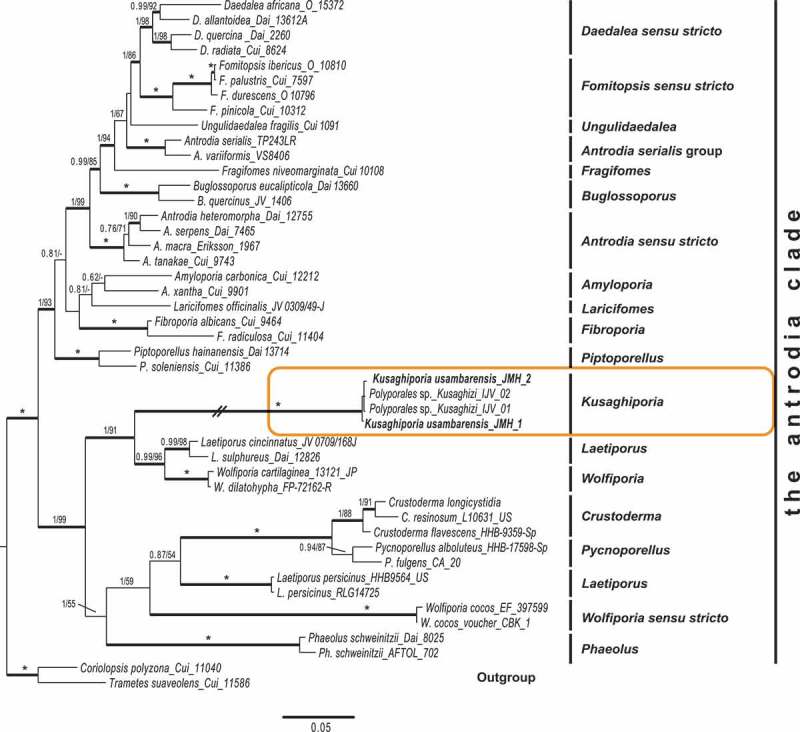
Phylogenetic relationships among *Kusaghiporia usambarensis* and allied taxa in the “antrodia clade”, based on Bayesian and ML analyses of concatenated nrLSU, nrSSU, RPB1 and TEF1 datasets. The tree was rooted using two species from the “core polyporoid clade” (*Coriolopsis polyzona* and *Trametes suaveolens*). The two support values associated with each internal branch correspond to PPs and MLbs proportions, respectively. Branches in bold indicate a support of PP ≥ 0.95 and MLbs ≥ 70%. An asterisk on a bold branch indicates that this node has a support of PP = 1.0 and MLbs = 100. The branch with double-slash is shortened. Clade names follow Han et al. (2016).

Analyses were based on a total of 209 sequences representing 201 species of *Polyporales*, with two russuloid species as out-group. The phylogeny of the *Polyporales* and the position of the “Kusaghizi” was inferred from four datasets: 36 nrLSU sequences, 25 nrSSU sequences, 26 RPB2 sequences and 22 TEF1 sequences. The *Polyporales* concatenated dataset (Figure 1) contained 100 sequences of 34 nrLSU, 21 nrSSU, 23 RPB2 and 22 TEF1. Further analyses included members of the “antrodia clade” (Figure 2) containing 145 concatenated sequences of 46 nrLSU, 33 nrSSU, 32 RPB2 and 35 TEF1. Maximum likelihood and Bayesian analyses of these datasets were undertaken, first separately and then also of the concatenated dataset. The analysis of the concatenated *Polyporales* dataset retrieved a phylogeny with five distinct clades (Figure 1) in addition to *Stereum hirsutum* and *Heterobasidion annosum*, as outgroup. Clade annotations follow Zhao et al. (2015). *Kusaghiporia usambarensis* was found to belong in the “antrodia clade”. The annotation of the concatenated phylogeny of the “antrodia clade” (Figure 2) follows Han et al. (2016).

### Taxonomy

**Figure 3.**
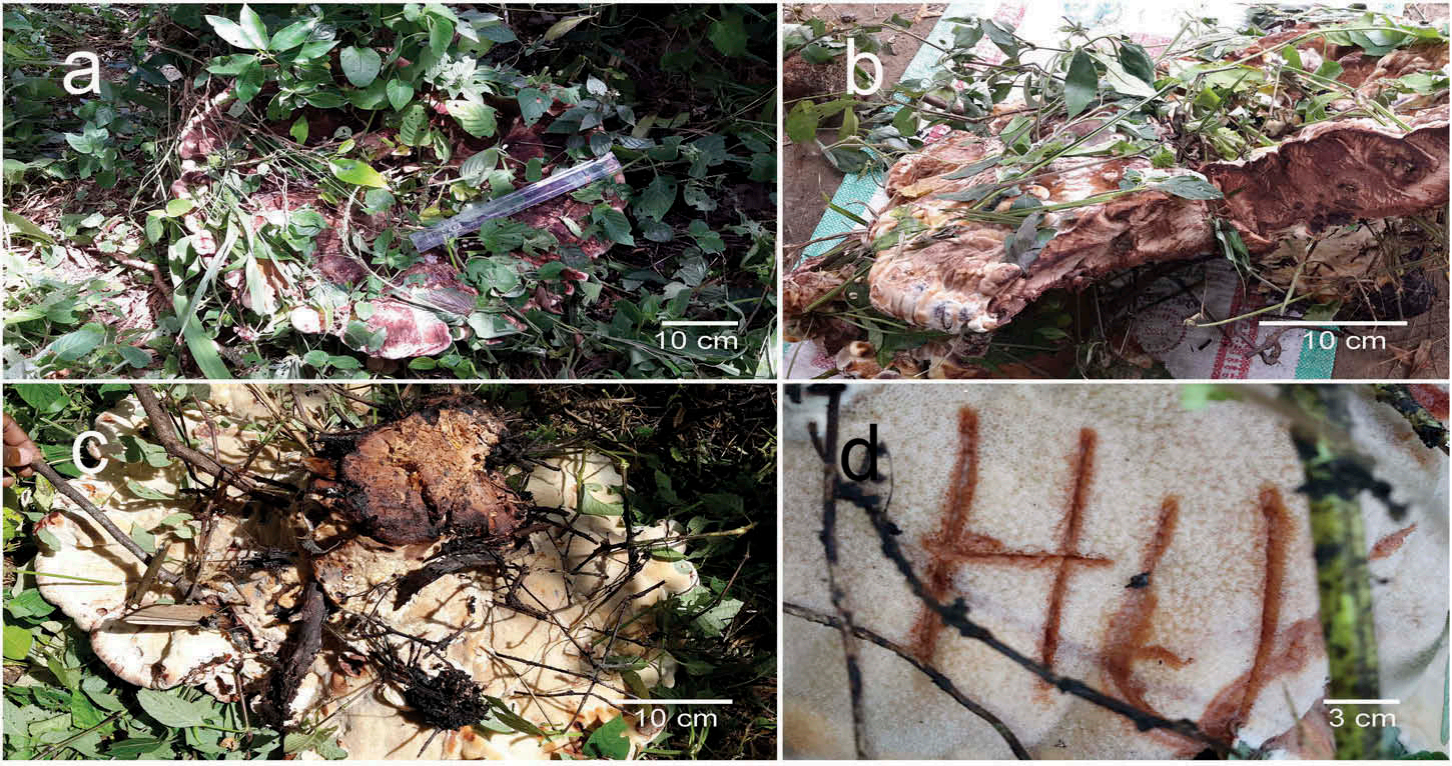
(a) Basidiocarp of *Kusaghiziporia usambarensis*(holotype). (b) Vertical section of basidiocarp. (c) Lower part of basidiocarp. (d) Bruise reaction, the creamy pores (5A2) turned brown (5D6).

**Figure 4.**
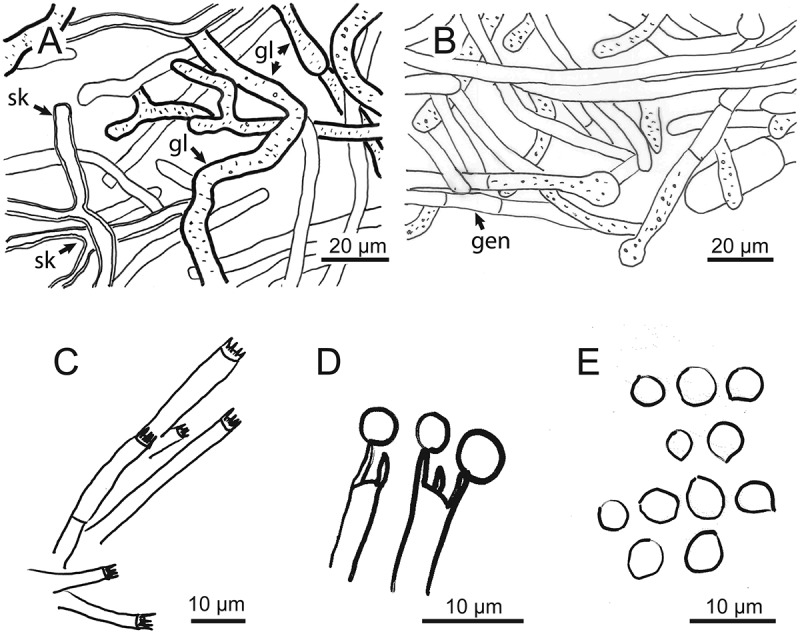
Microscopic structures of *Kusaghiziporia usambarensis* (holotype). (a) Skeletal hyphae (sk) with Y-shaped branches; gloeplerous hyphae (gl). (b) Septate generative hyphae (gen). (c) Basidia. (d) Basidia with spores attached to sterigmata. (e) Globular to subglobular basidiospores.

**Figure 5.**
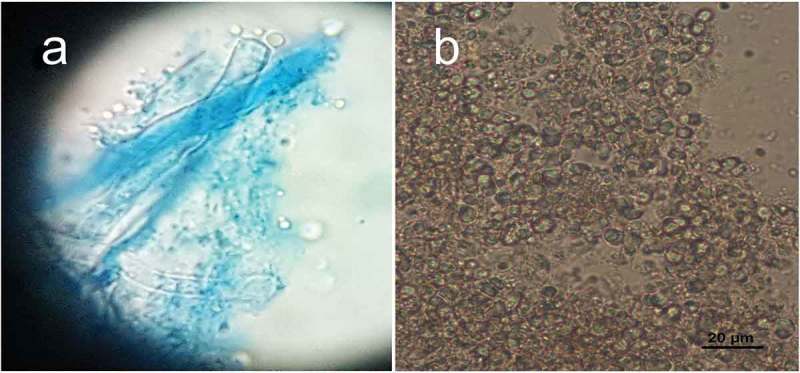
(a) Picture of basidia with spores attached to sterigmata. (b) Globular to subglobular basidiospores.

*Kusaghiporia usambarensis* Hussein J., Tibell S. & Tibuhwa, gen. et sp. nov. MycoBank no.: MB824538 [Figures 3, 4, 5.]

Basidioma annual, spathulate, viscid when young, at maturity saucer-shaped, bumpy, and with a spongy surface. Upper surface mottled dark brown with creamy patches. Hyphal system dimitic, with generative and skeletal hyphae. Gloeplerous hyphae present.

#### *Holotype* TANZANIA

Korogwe district, Tanga, Bungu, 18 February 2016, J. Hussein 01/16 (UPS); GenBank MH010044, MH010046, MH048871, MH048870.

#### Additional material examined

TANZANIA, Korogwe district, Tanga, Buti, 21 March 2017, J. Hussein 01/17 (UPS); GenBank MH010045, MH048869; Tanga, Makuri, 21 March 2017, J. Hussein 02/17 (UPS).

#### Etymology

*Kusaghiporia* refers to the sambaa name of the mushroom “Kusaghizi”, which means the collector or accumulator, and –poria (Lat.): with pores; *usambarensis* (Lat.): referring to the Usambara mountain range.

#### Fruitbody

Basidioma annual, spathulate, when young viscid, when mature depressed saucer-shaped, up to 60 cm in diameter with an uneven, velvety surface, surface dark brown at the centre (5E8), eroded (wrinkled), dark brown (5E8) to creamy (5A2); basidioma margin fleshy, up to 6-cm thick, dark brown (5E6) with pale brown (5C3) stripes. Stipe central, c. 12-cm high, c. 10 cm in diameter at the base, clavate, with creamy small dots (5A2), tough/woody, dark brown (5F8) in the inner part, without ring. The pores are creamy (5A2), turning brown (5D6) upon bruising. Cap in section dark brown (5E8) with creamy stripes (5A2), not changing upon exposure to air. Spore print whitish to creamy (5A2).

#### Hyphal structure

Hyphal system dimitic; generative hyphae with simple septa, hyaline, thin-walled 2.7–10.9 µm in diam (Figure 4(b)); skeletal hyphae hyaline, thick-walled, with Y-shaped branches 2.7–6.3 µm in diam (Figure 4(a)); gloeplerous hyphae present 3.6–11.8 µm in diam (Figure 4(a)).

#### Basidia and basidiospores

Basidia thin-walled, hyaline, tetrasterigmatic, 2.7–5.4 µm in diam (Figures 4(c, d) and 5(a)). Basidiospores hyaline, globose to subglobose, thin-walled, smooth, IKI-, 2.7–8.1 × 2.7–7.2 µm, *A*_L_ = 5.9 µm, *A*_W_ = 5.7 µm, *Q* = 1.04 (*n* = 40/1) (Figures 4(d, e) and 5(b)).

#### Rot type

Brown rot.

#### Host

Found growing at the base of the trees *Maesopsis eminii* and *Ficus natalensis*.

## Discussion

In earlier studies (Binder et al. 2013; Zhao et al. 2015), seven clades were found in *Polyporales*: the “core polyporoid clade”; the “residual polyporoid clade”; the “antrodia clade”; the “gelatoporia clade”; the “phlebioid clade”; the “tyromyces clade” and the “fragiliporia clade”. Justo et al. (2017) recognised six clades, excluding the “fragiliporia clade” reported by Zhao et al. (2015). We found *Kusaghiporia* to be nested within the “antrodia clade” in all analyses; concatenated dataset (Figure 1), and nrLSU, nrSSU, TEF1, RPB2 (Fig S1A, S1B, S1C; S1D). Analyses of *Kusaghiporia* and related taxa in the “antrodia clade” grouped *Kusaghiporia* with *Laetiporus* and *Wolfiporia*, a clade receiving strong support (Figure 2; 1 PP, 91% MLbs). Despite the strong support of *Laetiporaceae* Jülich (Figure 2) *K. usambarensis, L. persicinus, W. cocos, Phaoelus*, and *Crustoderma* together with *Pycnoporellus* displayed long branches indicating a high genetic divergence. A high genetic divergence of *L. persicinus* has previously been reported (Binder et al. 2013; Ortiz-Santana et al. 2013; Han et al. 2016; Justo et al. 2017). Lindner and Banik (2008) suggested placing *L. persicinus* in a separate genus due to its genetic remoteness as compared to other species of *Laetiporus*. Justo et al. (2017) suggested further studies to be needed for the delimitation of *Wolfiporia* and *Laetiporus*. However, a detailed discussion of *L. persicinus* and *W. cocos* is beyond the scope of this study.

*Kusaghiporia usambarensis* is morphologically similar to *Crustoderma, Pycnoporellus, Phaeolus, Wolfiporia and Laetiporus*, insofar that they all have hyphae with simple septa, produce annual polyporoid fruiting bodies with hyaline spores and cause brown rots (Lindner and Banik 2008). *Crustoderma* (Eriksson and Ryvarden 1975) differs from *K. usambarensis* in having resupinate basidio carps and a monomitic hyphal system. *Pycnoporellus* (Ryvarden and Melo 2014) differs from *K. usambarensis* in having yellow to orange basidiocarps and a monomitic hyphal system. *Phaeolus* (Lindner and Banik 2008) differs from *K. usambarensis* in having a monomitic hyphal system and producing hymenial cystidia. *Wolfiporia* and *Laetiporus*, like *K. usambarensis*, have dimitic hyphal systems. *Wolfiporia*, however, has resupinate basidiocarps and oblong-ellipsoid basidiospores (Zmitrovich et al. 2006). With the exception of *L. persicinus*, other *Laetiporus* species produce brightly coloured basidiocarps (Lindner and Banik 2008). *Kusaghiporia usambarensis* is different from *L. persicinus* in basidioma morphology (up to 60 cm) and the basiodiospores being globose to subglobose, while broadly ovoid in *L. persicinus*.

The BLAST results from GeneBank (NCBI, from 2017-10-16), using blastn with the program “discontiguous megablast” (for cross-species comparison, searching with coding sequences) with *Kusaghiporia* sequences, showed a highest sequence similarity for all four genes with *Laetiporus sulphureus*. Based on RPB2 and TEF1 they were: Query cover 99% and Ident. 87%, and Query cover 99%, Ident. 84% respectively. For nrSSU the highest similarity has a Query cover of 91% and Ident. 87%; while for nrLSU the Query cover was 100% and the Ident. 86%. Among the five top scores “*Polyporales* sp. Kusaghizi”, voucher IJV40-2 was found: Query cover only 60% and Ident. 100%. In our opinion the genetic isolation of *K. usambarensis* as compared to *Laetiporus* justifies the proposal of a new genus to accommodate the species investigated. The monophyly and strong support of the clade containing *K. usambarensis, Laetiporus, Wolfiporia, Crustoderma, Pycnoporellus*, and *Phaeolus* as shown in our phylogeny (Figure 2), also justifies the incorporation of *K. usambarensis* in *Laetiporaceae*.

## Conclusion

The new genus *Kusaghiporia* was described based on morphological characters and phylogenetic analyses based on concatenated sequence data from four genes. *Kusaghiporia* produces large fruit bodies. Together with *Laetiporus, Pycnoporellus, Phaeolus*, and *Wolfiporia* it formed a strongly supported clade (Figure 1) belonging in *Laetiporaceae*, which is nested in the “antrodia clade”. *Kusaghiporia* is a resource in the local communities of the Usambaras, where it is collected and eaten.
