# Supplementary material for: Phylogenetic position and taxonomy of Kusaghiporia usambarensis gen. et sp. nov. (Polyporales)
Source: Mycology. 2018 Apr 15;9(2):136–44. doi: 10.1080/21501203.2018.1461142 (PMC6059158; doi:10.1080/21501203.2018.1461142)
Supplement: Supplemental Material [file TMYC_A_1461142_SM4804.zip › Supplimentary_Text.docx]

S1A. Phylogenetic relationships among *Kusaghiporiausambarensis*and allied taxa in *Polyporales*, based on a Bayesian analysis of nrLSU dataset. The tree was rooted using two species from Russulales (*Heterobasidionannosum*and*Stereumhirsutum*). The two support values associated with each internal branch correspond to PPs and MLbsproportions, respectively. Branches in bold indicate a support of PP ≥ 0.95 and MLbs≥ 70 %. An asterisk on a bold branchindicates that this node has a support of PP = 1.0 and MLbs = 100. The branch with double-slash is shortened.Clade names follow Zhao et al. (2015).

S1B.Phylogenetic relationships among *Kusaghiporiausambarensis*and allied taxa in *Polyporales*,based on a Bayesian analysis of nrSSU dataset. The tree was rooted using two species from Russulales (*Heterobasidionannosum*and*Stereumhirsutum*). The two support values associated with each internal branch correspond to PPs and MLbsproportions, respectively. Branches in bold indicate a support of PP ≥ 0.95 and MLbs≥ 70 %. An asterisk on a bold branchindicates that this node has a support of PP = 1.0 and MLbs = 100. The branch with double-slash is shortened.Clade names follow Zhao et al. (2015).

S1C.Phylogenetic relationships among *Kusaghiporiausambarensis*and allied taxa in *Polyporales*, based on a Bayesian analysis of TEF1 dataset. The tree was rooted using two species from Russulales (*Heterobasidionannosum*and*Stereumhirsutum*). The two support values associated with each internal branch correspond to PPs and MLbsproportions, respectively. Branches in bold indicate a support of PP ≥ 0.95 and MLbs≥ 70 %. An asterisk on a bold branchindicates that this node has a support of PP = 1.0 and MLbs = 100. The branch with double-slash is shortened.Clade names follow Zhao et al. (2015).

S1D.Phylogenetic relationships among *Kusaghiporiausambarensis*and allied taxa in Polyporales, based on a Bayesian analysis of RPB2 dataset. The tree was rooted using two species from Russulales (Heterobasidionannosum and Stereumhirsutum). The two support values associated with each internal branch correspond to PPs and MLbsproportions, respectively. Branches in bold indicate a support of PP ≥ 0.95 and MLbs≥ 70 %. An asterisk on a bold branchindicates that this node has a support of PP = 1.0 and MLbs = 100. The branch with double-slash is shortened.Clade names follow Zhao et al. (2015).
