# Supplementary figures and images for: Phylogenetic position and taxonomy of Kusaghiporia usambarensis gen. et sp. nov. (Polyporales)
Source: Mycology. 2018 Apr 15;9(2):136–44. doi: 10.1080/21501203.2018.1461142 (PMC6059158; doi:10.1080/21501203.2018.1461142)

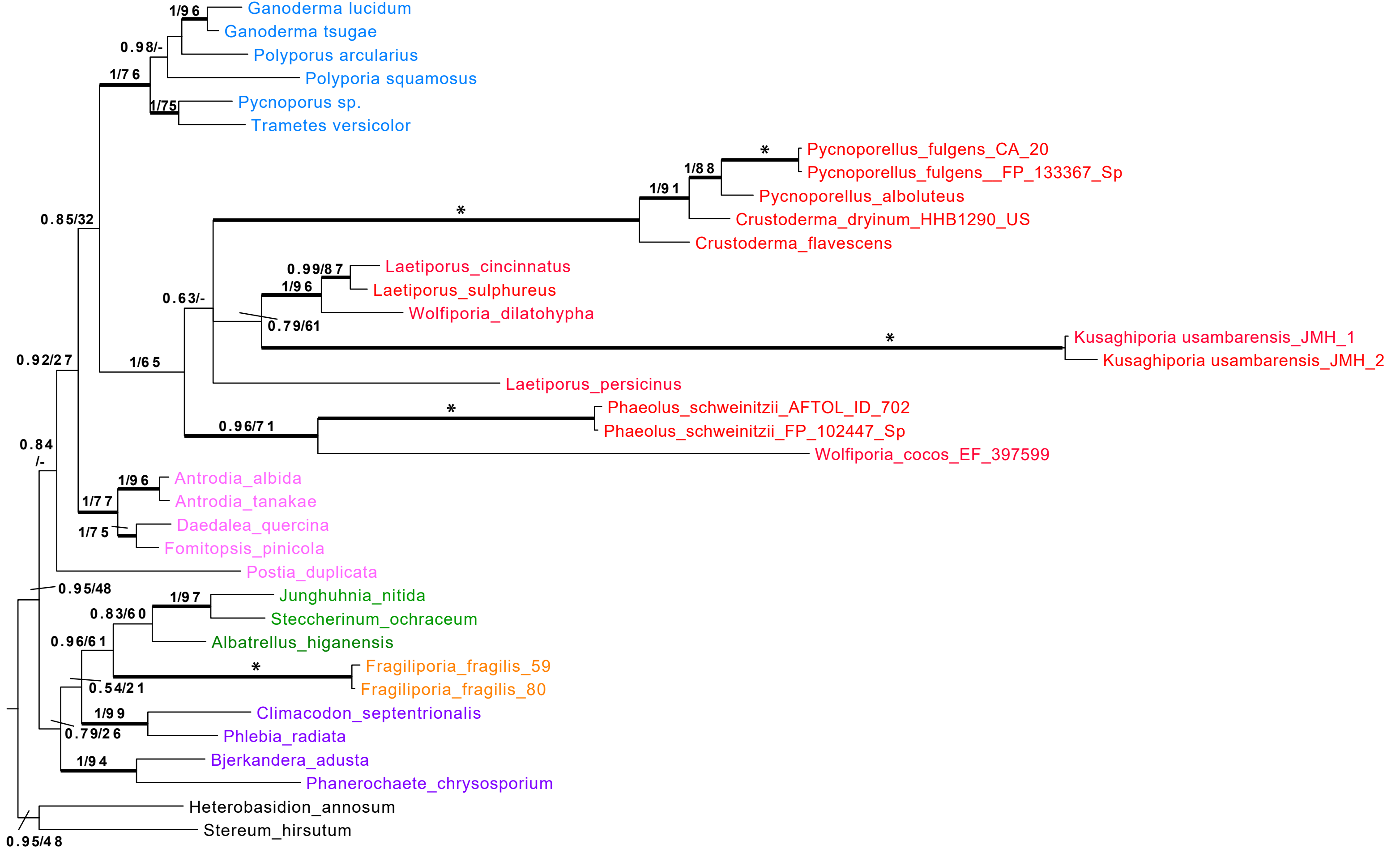

Supplement: Supplemental Material [file TMYC_A_1461142_SM4804.zip › BayesS1A(LSU).pdf]

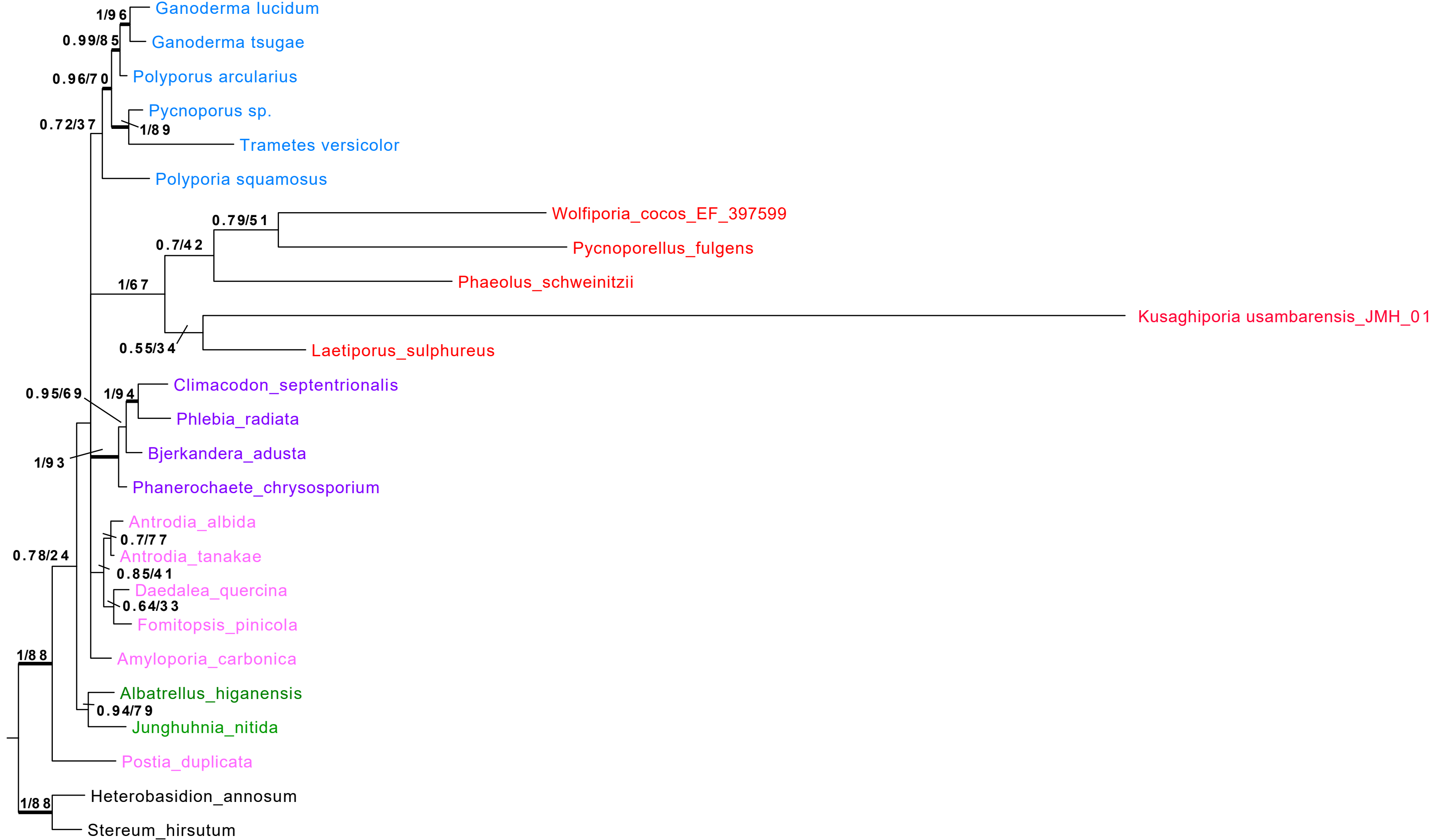

0.03

Supplement: Supplemental Material [file TMYC_A_1461142_SM4804.zip › BayesS1B(SSU).pdf]

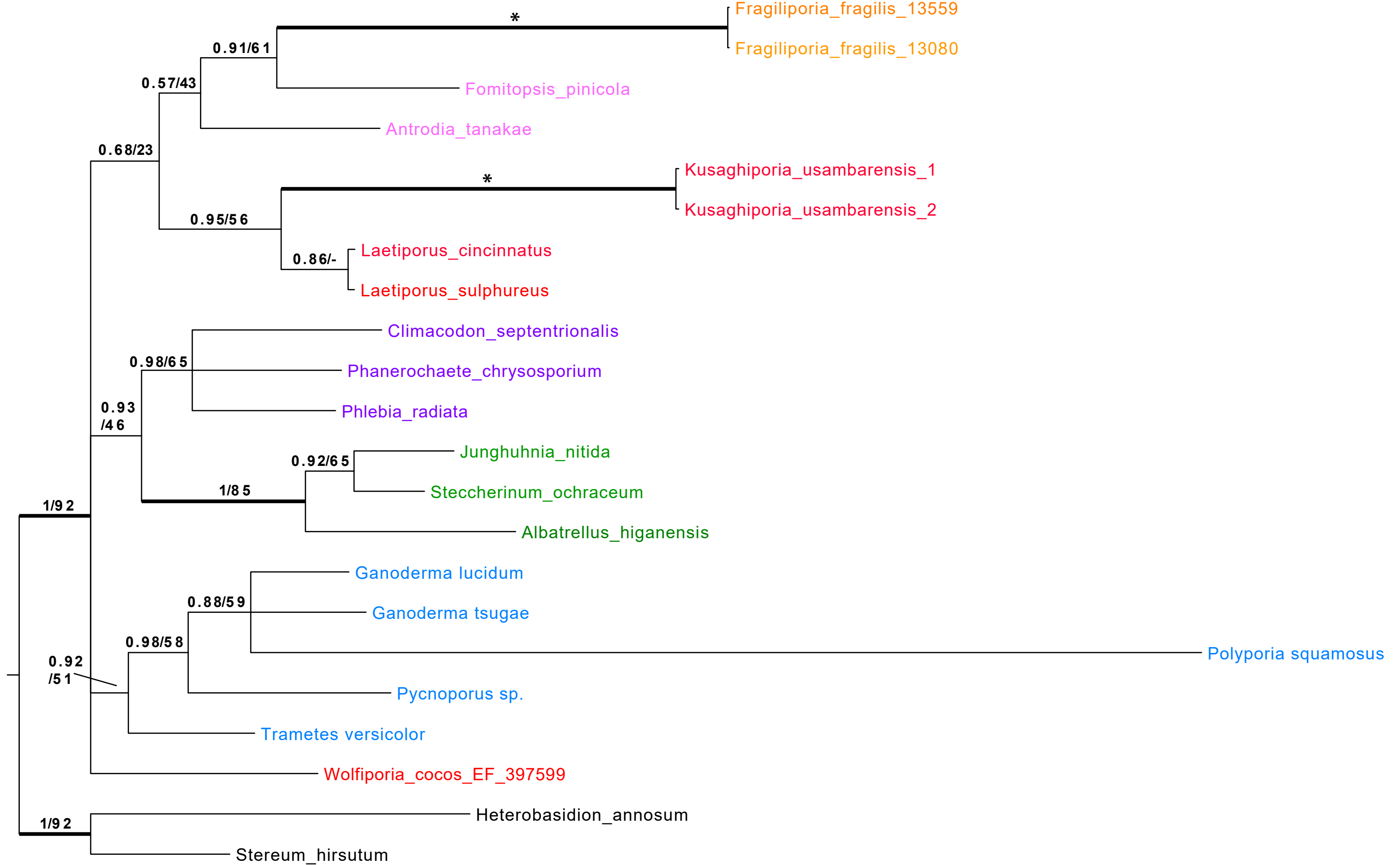

0.07

Supplement: Supplemental Material [file TMYC_A_1461142_SM4804.zip › BayesS1C(TEF1).pdf]

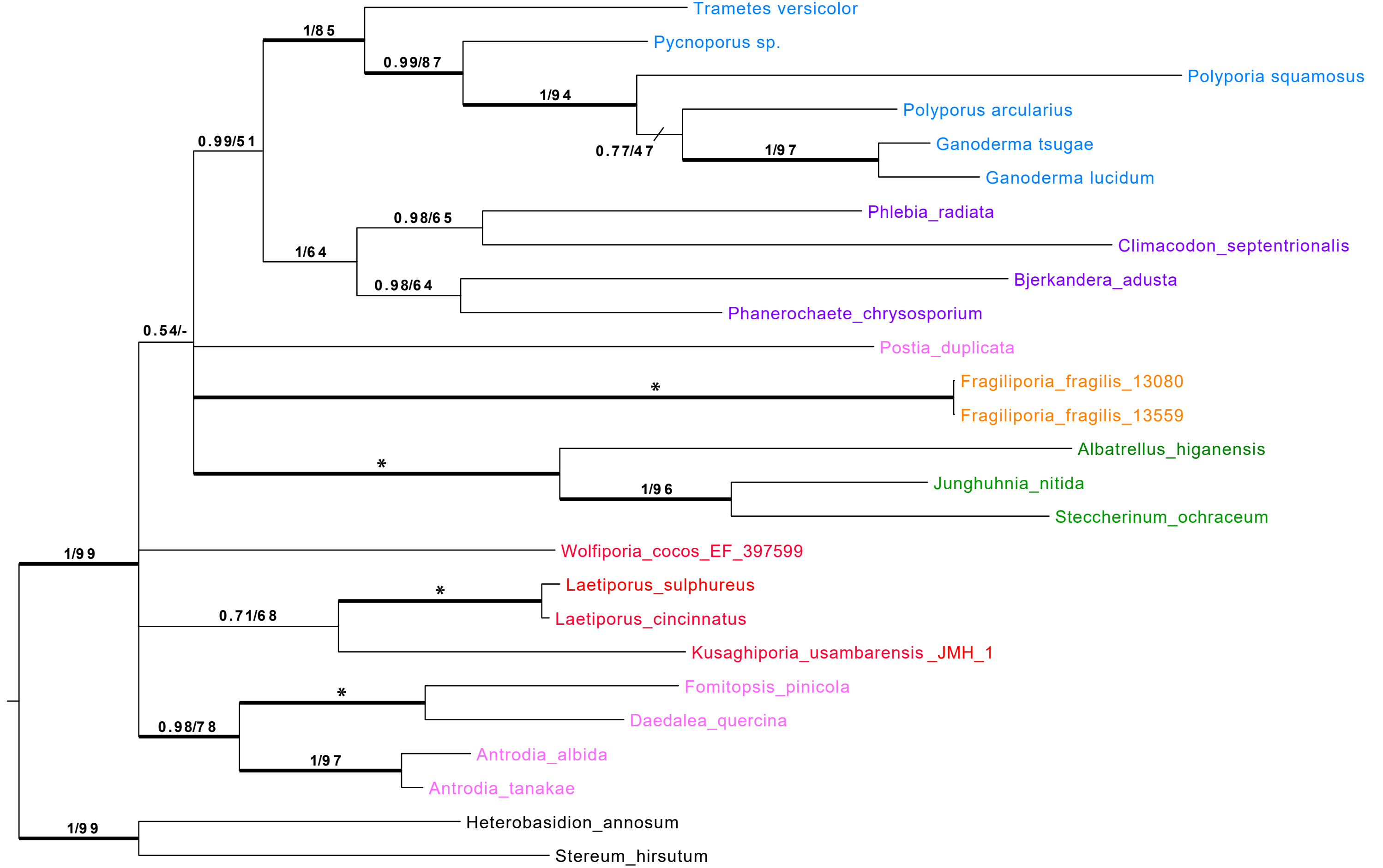

Supplement: Supplemental Material [file TMYC_A_1461142_SM4804.zip › BayesS1D(RPB2).pdf]
